# *CtARF4* Regulates Inflorescence Development Through Transcriptional Regulation of *CtMADS24* in Safflower

**DOI:** 10.3390/plants15071110

**Published:** 2026-04-03

**Authors:** Hengshuo Ge, Ping Xue, Yuting Liu, Xiaohui Pu, Weicong Zhu, Shiyu Luan, Qi Yang, Yuanyuan Dong

**Affiliations:** 1 College of Life Sciences, Jilin Agricultural University, Changchun 130118, China; 2 Department of Agriculture and Forestry Technology, Weinan Vocational and Technical College, Weinan 714000, China; lyxp001@163.com

**Keywords:** safflower, auxin response factors (ARFs), inflorescence development, organ specification, phylogenetic analysis, flowering

## Abstract

Safflower (*Carthamus tinctorius* L.) is a traditional economic crop in China valued for its medicinal petals and high-quality seed oil. Despite the importance of floret number and capitulum architecture for petal yield in safflower, the molecular regulators linking auxin signaling to inflorescence development in this species remain poorly understood. Auxin response factors (ARFs) are key transcriptional regulators mediating auxin-responsive gene expression and developmental processes, yet their functions in safflower inflorescence development have not been systematically investigated. In this study, we identified 25 *CtARF* genes from the safflower genome and classified them into five phylogenetic subfamilies. Cis-regulatory analysis predicted the presence of hormone- and development-related elements in *CtARF*-associated promoter regions. Expression profiling revealed that *CtARF4*, a member of the *CtARF III* subfamily, exhibits preferential expression during flower development. *CtARF4* was localized to the nucleus and shown to interact with the *CtMADS24* promoter and the Aux/IAA protein *CtIAA9* in heterologous systems. Transient overexpression of *CtARF4* increased floret number and length and promoted flowering, whereas virus-induced gene silencing resulted in opposite phenotypes. In addition, *CtARF4* perturbation was associated with a reduction in IAA content as measured by a kit-based assay. Collectively, these findings suggest that *CtARF4* functions as an auxin-responsive transcriptional regulator contributing to inflorescence development in safflower.

## 1. Introduction

A flower is composed of various tissues, including petals, sepals, stamens, and pistils. These parts are arranged and combined on the receptacle in specific patterns, forming diverse morphologies and structures to adapt to different pollination methods and reproductive strategies [1]. Flowering marks the transition of plants from vegetative to reproductive growth and involves numerous physiological processes, metabolic pathways, and gene regulatory mechanisms [2]. These mechanisms encompass intracellular and intercellular signal transduction cascades and the specific spatiotemporal expression of flowering genes [3,4]. Current research has revealed that hormones such as gibberellin play functional roles in the plant flowering process [5]. Meanwhile, although auxin has been found to regulate gene expression broadly and is involved in many processes of plant growth and development, its role in flowering and the associated molecular mechanisms remain poorly understood.

Studies on the model plant *Arabidopsis thaliana* have revealed a large number of genes regulated by auxin that play crucial roles in plant development [6,7,8]. Among these genes, members of the ARF family are particularly important in auxin signaling and regulating the expression of auxin-responsive genes [9,10]. Most ARFs contain three conserved functional domains: an N-terminal DNA-binding domain (DBD), a variable middle region (MR) that functions as either an activation domain (AD) or repression domain (RD), and a C-terminal dimerization domain (CTD) [11]. Current research has demonstrated that the expression of ARF genes in *Arabidopsis* is precisely and dynamically regulated across different developmental stages and tissues. For instance, AtARF2-4, AtARF3, AtARF5, and AtARF8 are essential for floral differentiation and development [12,13,14]. Recent studies on woodland strawberry (*Fragaria vesca*) have discovered that the FaARF4 gene can regulate inflorescence number and morphology during flower development. It has been further found that FaARF4 can bind to the promoter of FaAP1, a key regulator of flower development, thereby modulating FaAP1 expression and subsequently influencing floral development [15].

The plant hormone auxin participates in the regulation of nearly all developmental processes and controls gene expression through auxin signaling pathways. The ARF family is considered a core component of the auxin signaling pathway [16]. The CTD domain is involved in regulating ARF activity through dimerization with Aux/IAA family proteins and among ARFs themselves [17]. Under low auxin levels, ARFs are typically maintained in an inactive state by forming complexes with their repressors, the Aux/IAA proteins, thereby blocking the auxin signaling pathway. However, when auxin levels increase, Aux/IAA proteins can be directly recognized by the SCF (TIR1/AFB) ubiquitin ligase complex and subsequently degraded by the 26S proteasome, which relieves the repression of ARFs. Once freed from Aux/IAA inhibition, ARFs can activate or repress the expression of auxin-dependent genes. Thus, the Aux/IAA-ARF module governs various processes in plant growth and development, such as apical dominance, fruit development and ripening, as well as cell division, expansion, and differentiation [17].

Numerous studies suggest that the ARF family may display auxin response specificity, and different ARF members can elicit diverse auxin response reactions to appropriately initiate developmental processes [18,19]. The most straightforward evidence stems from the discoveries that many ARF genes operate as transcriptional activators, while others serve as repressors [20]. For example, MdARF13 in apple can suppress anthocyanin biosynthesis [21], whereas MdARF8 facilitates lateral root formation [22]. Presently, no research has explored the functions of ARF genes within the safflower species.

Recent studies have conducted genome-wide identification and expression profiling of the *ARF* gene family in safflower, revealing conserved sequence motifs, gene structural features, phylogenetic relationships, and expression patterns [23]. These investigations provide fundamental insights into the potential roles of the ARF gene family in floral development. However, comprehensive analyses of the expression profiles of the *CtARF* gene family across different tissues and developmental stages of the inflorescence remain limited. Furthermore, the regulatory mechanisms controlled by ARF transcription factors, particularly through the detailed characterization of promoter elements during various stages of flower development, have not been fully explored.

## 2. Results

### 2.1. Genome-Wide Identification and Promoter Cis-Element Analysis of CtARF Genes

**Figure 1:**
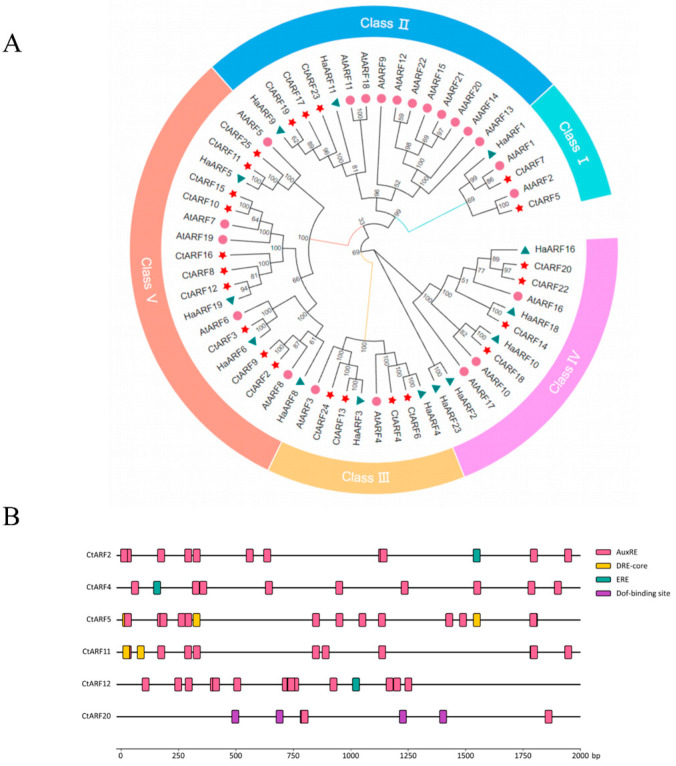
Phylogenetic analysis and promoter cis-element distribution of ARF genes: (**A**) Phylogenetic relationships of ARF proteins from *Carthamus tinctorius*, *Arabidopsis thaliana*, and *Helianthus annuus*. Ct, Ha, and At indicate safflower, sunflower, and *Arabidopsis*, respectively. The red pentagram represents safflower ARFs, the green triangle represents sunflower ARFs, and the pink circle represents *Arabidopsis* ARFs. A total of 61 ARF proteins (25 from safflower, 14 from sunflower, and 22 from *Arabidopsis*) were used to construct the phylogenetic tree. The ARF proteins were classified into five groups (I–V). Bootstrap values were calculated with 1000 replicates. (**B**) Distribution of major cis-acting elements in the promoter regions (2000 bp upstream) of six selected *CtARF* genes. The analyzed motifs include the auxin response element (AuxRE), DRE-core (dehydration-responsive element), ERE (ethylene-responsive element), and Dof-binding sites. The number and positional distribution of these motifs vary among promoters, with AuxRE being the most widely distributed element.

To identify ARF family genes in safflower (*Carthamus tinctorius* L.), coding sequences of ARF genes from *Arabidopsis thaliana* and sunflower (*Helianthus annuus*) were used as queries for the NCBI BLAST web server searches against the safflower genome. After removing redundant and incomplete sequences, a total of 25 *CtARF* genes were identified. Phylogenetic analysis classified these *CtARF* members into five subfamilies (Figure 1A). Within each subfamily, *CtARFs* clustered more closely with sunflower ARFs than with those from *Arabidopsis*, consistent with their closer evolutionary relationship within the Asteraceae lineage.

To identify potential target genes of *CtARF* transcription factors, we performed a genome-wide search for promoter regions bound by each of the 25 *CtARF* members using PlantTFDB (v5.0) [24] and JASPAR [25]. Among the 25 *CtARF* members, only six proteins, *CtARF2*, *CtARF4*, *CtARF5*, *CtARF11*, *CtARF12*, and *CtARF20*, were predicted to have identifiable target genes; the remaining 19 showed no detectable targets under the current criteria. These six were therefore selected for detailed analysis of their target promoter architectures.

As shown in Figure 1B, promoters targeted by different *CtARF* members exhibited distinct transcription factor-binding motifs. The auxin response element (AuxRE) was present across all six, with the highest density in *CtARF12*-targeted promoters and the lowest in *CtARF20*-targeted ones. DRE-core motifs were exclusive to *CtARF5*- and *CtARF11*-targeted promoters, while ERE motifs appeared in promoters targeted by *CtARF2*, *CtARF4* (including the *CtMADS24* promoter), and *CtARF12*. Dof-binding sites were uniquely enriched in *CtARF20*-targeted promoters. The identification of *CtMADS24* as a putative target of *CtARF4* guided subsequent experimental validation.

### 2.2. Expression Profiling Identifies CtARF4 as a Flower-Preferential Candidate

**Figure 2:**
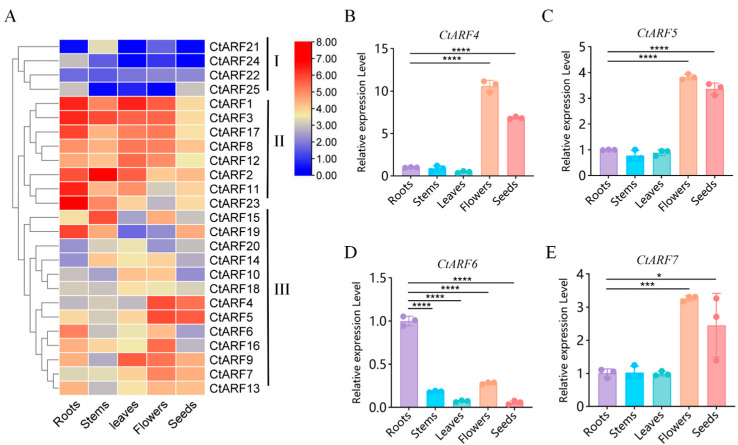
Expression analysis of *CtARF* genes in different tissues of safflower: (**A**) Hierarchical clustering heatmap depicting expression profiles of 25 *CtARF* genes across five tissues: roots, stems, leaves, flowers, and seeds. Genes are grouped into three classes (I–III) based on expression patterns. Color scale reflects transcript abundance from low (blue) to high (red). (**B**–**E**) Relative expression levels of representative Class III genes: *CtARF4* (**B**), *CtARF5* (**C**), *CtARF6* (**D**), and *CtARF7* (**E**) in roots, stems, leaves, flowers, and seeds. * *p* < 0.05, *** *p* < 0.001, **** *p* < 0.0001.

To examine the expression patterns of *CtARF* genes in safflower, RNA-seq data from five tissues (roots, stems, leaves, flowers, and seeds) were analyzed (Figure 2A). Based on hierarchical clustering of expression profiles, the 25 *CtARF* genes were classified into three expression classes.

*Class I* genes, including *CtARF21*, *CtARF24*, *CtARF22*, and *CtARF25*, exhibited consistently low transcript abundance across all examined tissues. *Class II* genes, such as *CtARF1*, *CtARF3*, *CtARF17*, *CtARF8*, *CtARF12*, *CtARF2*, *CtARF11*, and *CtARF23*, showed relatively higher expression levels in multiple tissues, with moderate to low expression in seeds. *Class III* genes displayed tissue-biased expression patterns. Among them, *CtARF4* and *CtARF5* showed higher transcript abundance in flowers, whereas *CtARF6* was predominantly expressed in roots (Figure 2A).

To validate the RNA-seq results, four representative genes (*CtARF4*, *CtARF5*, *CtARF6*, and *CtARF7*) were selected for qRT-PCR analysis. The qRT-PCR results were consistent with the transcriptomic data. *CtARF4* and *CtARF5* showed significantly higher expression levels in flowers compared with other tissues (Figure 2B,C). *CtARF6* exhibited the highest expression in roots, with moderate expression in flowers and seeds (Figure 2D). *CtARF7* displayed elevated expression in flowers and detectable expression in seeds (Figure 2E).

**Figure 3:**
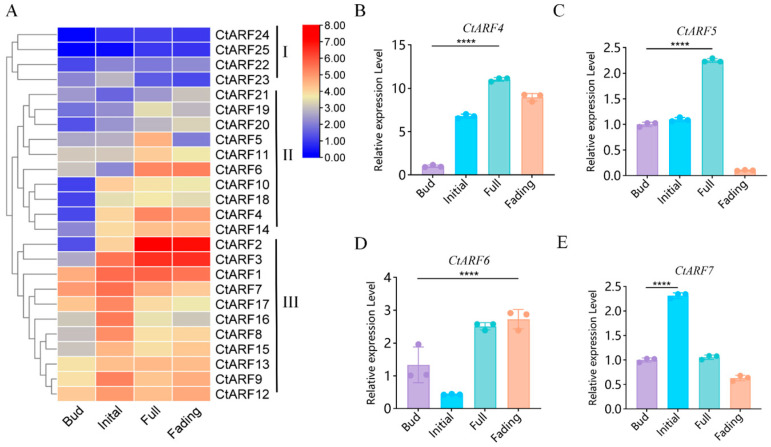
Expression analysis of *CtARF* genes during flower development stages in safflower: (**A**) Hierarchical clustering heatmap of *CtARF* gene expression across four developmental stages: Bud, Initial, Full, and Fading. Genes are clustered into three groups (I–III) based on expression similarity. The color scale represents transcript abundance from low (blue) to high (red). (**B**–**E**) Stage-specific expression patterns of selected *CtARF* genes: *CtARF4* (**B**), *CtARF5* (**C**), *CtARF6* (**D**), and *CtARF7* (**E**). **** *p* < 0.0001.

To investigate the expression dynamics of *CtARF* genes during flower development, transcriptomic data from four developmental stages (bud, initial flowering, full bloom, and fading) were analyzed (Figure 3A). Hierarchical clustering grouped the *CtARF* genes into three classes based on their temporal expression patterns.

*Class I* genes maintained low transcript levels throughout all developmental stages. Class II genes showed increased expression from the initial flowering stage to full bloom, with some remaining elevated during the fading stage. *Class III* genes exhibited higher expression at the bud or initial flowering stages, followed by a gradual decrease during later stages (Figure 3A).

Four *CtARF* genes (*CtARF4*, *CtARF5*, *CtARF6*, and *CtARF7*) with relatively high expression during flower development were further examined by qRT-PCR. *CtARF4* and *CtARF5* showed increased expression from the initial flowering stage to full bloom, followed by a decrease at the fading stage (Figure 3B,C). *CtARF6* showed lower expression at the initial flowering stage and higher expression at the full bloom and fading stages (Figure 3D). *CtARF7* reached its highest expression at the initial flowering stage and declined thereafter (Figure 3E).

*CtARF4* displayed a flower-preferential expression pattern and was upregulated during the bud-to-bloom transition, and was therefore selected for in-depth functional analysis.

### 2.3. Subcellular Localization of CtARF4

To investigate the potential function of *CtARF4* in flower development, we first examined its subcellular localization. Bioinformatics prediction using the Softberry online tool indicated the presence of a nuclear localization signal (NLS) in the *CtARF4* protein, suggesting its nuclear localization. To experimentally verify this prediction, we constructed a 35S:*CtARF4*-DsRed fusion vector and introduced it into tobacco leaf epidermal cells, with the 35S:DsRed vector serving as a control. The bacterial suspensions containing the recombinant vector and the 35S:DsRed vector were then injected into tobacco leaf cells for experimental verification.

**Figure 4:**
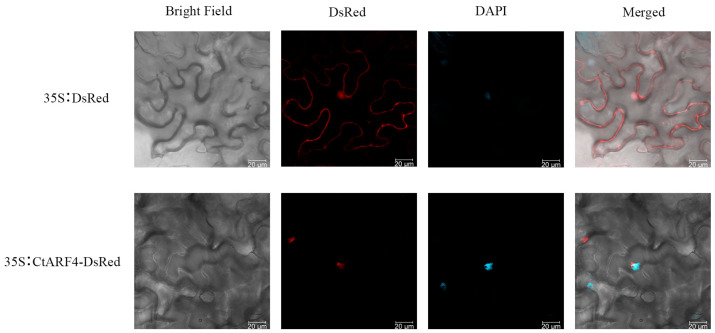
Subcellular localization of *CtARF4* using a transient expression system in tobacco leaves. The *CtARF4*-Dsred fusion construct was localized in the nucleus. The fluorescence signal was detected by a laser scanning confocal microscope. DsRed represents the fluorescence of red fluorescent protein. Scale bar = 50 μm.

Confocal laser scanning microscopy observations at 48 h post-transformation revealed distinct localization patterns for the two constructs (Figure 4). The red fluorescence of the free DsRed protein from the control vector was distributed throughout the entire cell, including both the cytoplasm and the nucleus. In contrast, the fluorescence signal of the *CtARF4*-DsRed fusion protein was exclusively concentrated within the nucleus, closely aligning with the nuclear morphology.

This result experimentally confirms the bioinformatics prediction and demonstrates that *CtARF4* is a nuclear-localized protein. This localization is consistent with its putative function as a transcription factor that regulates downstream gene expression by binding to promoter elements in the nucleus.

### 2.4. Functional Analysis of CtARF4 in Safflower Flower Development

To investigate the role of *CtARF4* in safflower flower development, we generated overexpression (*CtARF4*-OE) and virus-induced gene silencing (*CtARF4*-VIGS) lines. qRT–PCR analysis confirmed that *CtARF4* transcript levels were markedly increased in three independent OE lines and significantly reduced in three VIGS lines compared with wild-type (WT) plants (Supplementary Figure S1). These lines were therefore selected for detailed phenotypic and molecular analyses.

**Figure 5:**
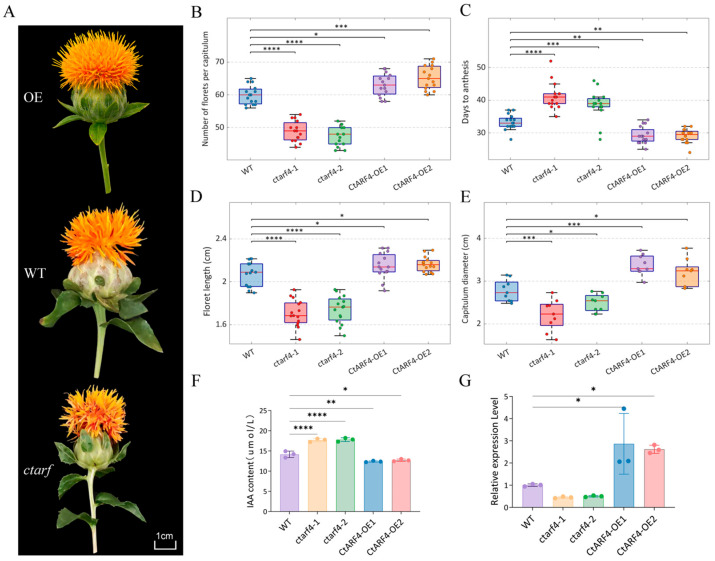
Functional analysis of *CtARF4* in safflower floral development: (**A**) Representative floral phenotypes of WT, *CtARF4*-overexpressing (*CtARF4*-OE), and *CtARF4*-silenced plants. Scale bar = 1 cm. (**B**) Number of florets per capitulum. (**C**) Days to anthesis. (**D**) Floret length. (**E**) Capitulum diameter. (**F**) Endogenous IAA content (ng g^−1^ FW). (**G**) Relative expression of *CtMADS24*. Data in (**B**–**E**) are shown as boxplots, n=15. Data in (**F**,**G**) are presented as mean ± SD, n=3. Asterisks above the boxes indicate statistically significant differences (*p* < 0.05, one-way ANOVA followed by Tukey’s test). * *p* < 0.05, ** *p* < 0.01, *** *p* < 0.001, **** *p* < 0.0001.

Relative to wild-type (WT) plants, *CtARF4*-overexpressing (*CtARF4*-OE) lines developed noticeably larger capitula, elongated florets, enhanced petal pigmentation, and showed an earlier onset of flowering. In contrast, *CtARF4*-silenced plants exhibited smaller capitula, shortened florets, incomplete flower opening, and visibly reduced pigmentation (Figure 5A and Supplementary Figure S2).

Quantitative measurements further demonstrated that *CtARF4*-OE plants had a significantly higher number of florets per capitulum (7.04%; Figure 5B), advanced anthesis by approximately 4 days (Figure 5C), increased floret length (3.13%; Figure 5D), and a larger capitulum diameter (14.3%; Figure 5E) compared with WT plants. Conversely, *CtARF4*-silenced plants showed a marked decrease in floret number (19.60%), delayed flowering by approximately 7 days, reduced floret length (16.25%), and a smaller capitulum diameter (21.1%) relative to WT plants (Figure 5B–E).

Endogenous auxin quantification revealed that indole-3-acetic acid (IAA) levels were significantly elevated in flowers of *CtARF4*-silenced plants, whereas *CtARF4*-OE lines displayed a moderate but statistically significant reduction in IAA content compared with WT plants (Figure 5F).

Furthermore, expression analysis showed that *CtMADS24* transcript levels were significantly down-regulated in *CtARF4*-silenced plants but up-regulated in *CtARF4*-OE lines (Figure 5G).

### 2.5. Transcriptional Regulation of the CtMADS24 Promoter by CtARF4 in Safflower

**Figure 6:**
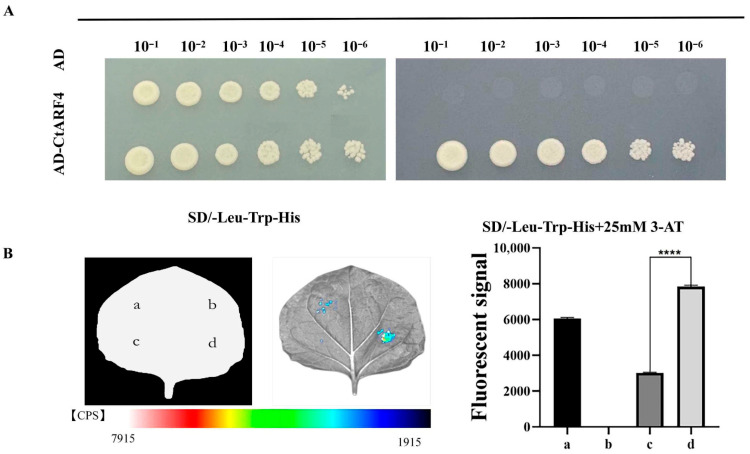
*CtARF4* binds to the *CtMADS24* promoter and activates its transcription: (**A**) Yeast one-hybrid assay showing the interaction between *CtARF4* and the *CtMADS24* promoter (proCtMADS24). Yeast cells co-transformed with the pHIS2-proCtMADS24 bait vector and either the empty pGADT7 (AD) vector or the pGADT7-*CtARF4* (AD-*CtARF4*) prey vector were serially diluted (10^−1^ to 10^−6^) and spotted onto SD/–Leu/–Trp (**left**) or SD/–Leu/–Trp/–His medium supplemented with 25 mM 3-AT (**right**). (**B**) Dual-luciferase (LUC) reporter assay assessing the transcriptional activation of proCtMADS24 by *CtARF4* in *N. benthamiana* leaves. (**Left**) Schematic diagram of the effector and reporter constructs used in the assay. a: Positive; b: pGreenII-0800-Luc + pGreenII-62-SK; c: pGreenII-0800-Luc-proCtMADS24 + pGreenII-62-SK; d: pGreenII-0800-Luc-proCtMADS24 + pGreenII62-SK-*CtARF4*. (**Middle**) Chemiluminescence images of representative leaves, with a color scale indicating signal intensity. (**Right**) Quantitative analysis of LUC relative luciferase activity. **** *p* < 0.0001.

To examine whether *CtARF4* is capable of recognizing the *CtMADS24* promoter, a yeast one-hybrid (Y1H) assay was performed. Yeast cells co-transformed with pGADT7-*CtARF4* and pHIS2-proCtMADS24 were able to grow on SD/–Leu/–Trp/–His medium supplemented with 25 mM 3-AT, whereas cells carrying the empty pGADT7 vector failed to grow under the same selective conditions (Figure 6A). These results indicate a sequence-dependent interaction between *CtARF4* and the *CtMADS24* promoter fragment in yeast.

To further assess whether *CtARF4* modulates *CtMADS24* promoter activity in a plant system, a transient dual-luciferase (Dual-LUC) assay was conducted in *Nicotiana benthamiana* leaves. Co-expression of *CtARF4* with the proCtMADS24:LUC reporter significantly increased the LUC/REN ratio compared with the empty effector control (Figure 6B), indicating that *CtARF4* enhances *CtMADS24* promoter activity in a heterologous plant context.

Together, these assays demonstrate that *CtARF4* can interact with the *CtMADS24* promoter and activate its transcriptional activity in heterologous systems, supporting *CtMADS24* as a likely downstream target of *CtARF4*-mediated transcriptional regulation.

### 2.6. CtARF4 Protein Interaction with CtIAA9

**Figure 7:**
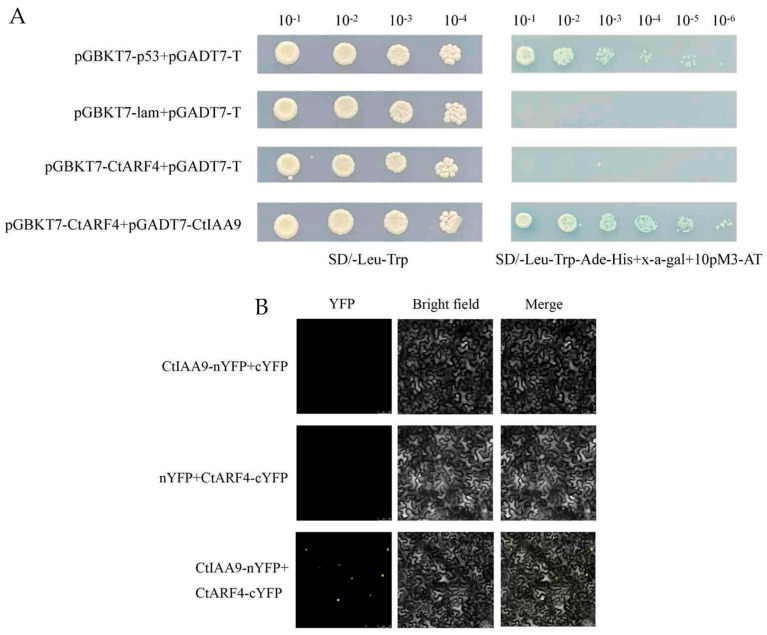
Protein–protein interaction between *CtARF4* and *CtIAA9*. (**A**) Yeast two-hybrid (Y2H) assay. The combination pGBKT7-*CtARF4* + pGADT7-*CtIAA9* grew and turned blue on the selective medium. The p53 + pGADT7-T and Lam + pGADT7-T pairs served as positive and negative controls, respectively. (**B**) Bimolecular fluorescence complementation (BiFC) assay in *N. benthamiana* epidermal cells. YFP fluorescence signal, bright-field image, and the merged view are shown. Scale bar = 50 μm.

To investigate whether *CtARF4* participates in auxin signaling through protein interaction, we first analyzed its potential binding partners using the STRING protein interaction database. The prediction suggested a potential interaction between *CtARF4* and *CtIAA9*, a member of the IAA family in safflower. To validate this, a yeast two-hybrid (Y2H) assay was performed. The result confirmed a specific interaction between *CtARF4* and *CtIAA9* in yeast cells (Figure 7A).

For further verification in plant cells, we conducted a bimolecular fluorescence complementation (BiFC) assay. The coding sequences of *CtARF4* and *CtIAA9* were fused to the C-terminal (cYFP) and N-terminal (nYFP) fragments of yellow fluorescent protein, respectively, and co-expressed in Nicotiana benthamiana leaves. Confocal microscopy observation revealed the reconstitution of YFP fluorescence predominantly in the nucleus (Figure 7B), indicating that *CtARF4* and *CtIAA9* interact directly within the nuclear compartment. This subcellular localization of their interaction is consistent with the established model of nuclear auxin signaling transduction. We also examined *CtMADS24* and *CtIAA9* expression using the same RNA-seq dataset. As shown in Supplementary Figure S3, *CtMADS24* expression increased during flower development and peaked at the fading stage, while *CtIAA9* showed the highest expression at the fading stage.

### 2.7. Expression Analysis of Inflorescence-Related Genes Under Auxin Treatment

**Figure 8:**
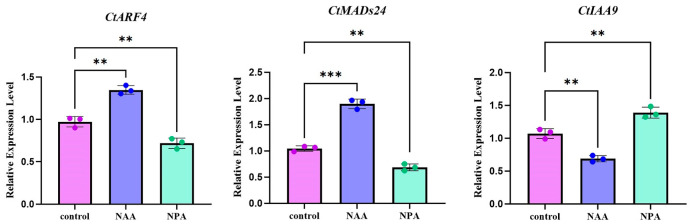
Relative expression levels of *CtARF4*, *CtMADS24*, and *CtIAA9* in response to treatments with NAA and NPA. Vertical bars represent the SDs (*n*  =  3). ** *p*  <  0.01, *** *p*  <  0.001.

To examine the expression responses of *CtARF4*, *CtMADS24*, and *CtIAA9* to auxin-related treatments, safflower flower buds were treated with water (control), 50 μM NAA, or 50 μM NPA. Transcript levels of the three genes were subsequently analyzed by qRT-PCR. *CtIAA9* transcript levels were reduced following NAA treatment and increased after NPA treatment compared with the control (Figure 8). In contrast, the transcript levels of *CtARF4* and *CtMADS24* were increased by NAA treatment and decreased by NPA treatment (Figure 8).

## 3. Discussion

Flower development involves reproductive growth, flowering time regulation, and vegetative growth monitoring, which represent only a small fraction of the many roles ARF genes play in flowering plants. As a crucial component of the auxin signaling pathway, ARFs are key transcription factors in the auxin signaling pathway and are known to bind auxin-responsive elements in the promoters of downstream genes [26]. Previous studies have shown that the ARF family plays a vital role in plant growth, development, hormone response, and stress response [27,28], making it a key gene family for understanding plant biology. Understanding the dynamics of this gene family provides an important resource for enhancing yield through varietal improvement. However, no studies have been reported on the role of ARF transcription factors in safflower during inflorescence growth and development.

In this study, a total of 25 *CtARF* genes were identified from the safflower genome after excluding missing and incomplete sequences. The number of *ARF* genes varies among different species: safflower (25), sunflower (23), and *Arabidopsis thaliana* (22 functional genes and 1 pseudogene) [11]. According to the classification system established for *Arabidopsis thaliana* [29], *CtARF* genes can be divided into five subfamilies, with *CtARF4* belonging to Class III. Further analysis revealed that many *CtARFs* cluster within the same clades as functionally characterized *AtARFs*. For instance, in the phylogenetic tree, *AtARF4* has been reported to participate in the regulation of flowering in several plant species [15].

This study provides a motif-based prediction of transcription factor (TF)–promoter interactions in safflower. In this prediction, a limited number of TFs, particularly *CtARF4*, showed high connectivity with multiple putative target promoters, including the promoter of *CtMADS24*. This computational result is consistent with our experimental observations that *CtARF4* is associated with key floral traits and may participate in transcriptional regulation during safflower flower development. In addition, the highly specific distribution of predicted TF-binding motifs among promoters may indicate a modular regulatory architecture that contributes to fine-tuning gene expression in safflower. This observation complements previous genome-wide analyses of MADS-box and R2R3-MYB transcription factor families reported in safflower [30]. Although this computational framework provides a systematic overview of putative regulatory relationships, these predicted interactions require further experimental validation.

*CtMADS24* belongs to the AP1 subfamily of MADS-box genes, and AP1-like genes have been reported to regulate flowering and floral organ development in *Arabidopsis* and other species [28]. This prior knowledge supports the potential relevance of *CtMADS24* in safflower floral development. Yeast one-hybrid (Y1H) and dual-luciferase reporter assays indicated that *CtARF4* can bind to the *CtMADS24* promoter and enhance its transcriptional activity, consistent with ARF-mediated promoter regulation reported in other plant species [15]. Together with the present findings, this supports a model in which *CtARF4* contributes to inflorescence development, at least in part, through transcriptional regulation of *CtMADS24*.

*ARF* genes are key components within the Aux/IAA–ARF module and have been identified and studied in numerous plant species. Subcellular localization analysis revealed that *CtARF4* is localized in the nucleus in *Nicotiana benthamiana*, consistent with findings for *ARF* genes in other species [10]. Moreover, qRT–PCR analysis showed that *CtARF4* transcript levels increased after NAA treatment but decreased following NPA treatment [15]. Together, these responses suggest that *CtARF4* may be involved in auxin-responsive regulation. Auxin (IAA) regulates plant growth and development through signal transduction, and ARF proteins act as central transcriptional regulators in this pathway; ARF gene expression can itself be influenced by auxin levels [17]. Similarly, ARF4 expression has been reported to respond to auxin in *Arabidopsis thaliana* and *Medicago truncatula* [31].

To examine whether *CtARF4* targets flowering-related genes, yeast one-hybrid and dual-luciferase assays indicated that *CtARF4* is capable of binding to the *CtMADS24* promoter and enhancing its transcriptional activity under experimental conditions. Comparable ARF4–AP1 promoter associations have been reported in strawberry, where FveARF4 binds to the FveAP1 promoter [15]. AP1-like genes are widely recognized for their roles in floral meristem identity and floral organ development across plant species [32,33,34]. In addition, protein–protein interaction prediction using STRING suggested *CtIAA9* as a potential interactor of *CtARF4*. Yeast two-hybrid and bimolecular fluorescence complementation (BiFC) assays further supported a physical interaction between *CtARF4* and *CtIAA9*, consistent with the conserved Aux/IAA–ARF module described in other species [21]. Although the BiFC experiments did not include a nuclear marker, the nuclear localization of *CtARF4* was confirmed by DAPI co-staining in subcellular localization assays (Figure 4), supporting the nuclear context of these interactions.

To explore how *CtARF4* regulates flower development in safflower, we employed virus-induced gene silencing (VIGS) [35] to suppress *CtARF4* expression, generating gene-silenced plants, and obtained transient overexpression lines of *CtARF4* driven by the CaMV35S promoter. Phenotypic comparisons among these plants and the control group revealed that 35S:*CtARF4* plants exhibited accelerated development compared to both the control and TRV2:*CtARF4* groups. The number and length of safflower florets were also greater in the overexpression lines, accompanied by an earlier flowering time. In contrast, TRV2:*CtARF4* plants flowered significantly later than the control, produced fewer florets, and had shorter floret lengths. Endogenous indole-3-acetic acid (IAA) content measurements showed that IAA levels were lower in 35S:*CtARF4* plants and higher in TRV2:*CtARF4* plants relative to the control. These results demonstrate an association between *CtARF4* expression and safflower developmental processes. Moreover, silencing *CtARF4* may delay flower formation and prolong the vegetative growth phase, leading to upregulation of endogenous auxin levels, whereas the opposite effect is observed in overexpression lines; these associations also need additional experimental support to confirm causality.

Overall, this study provides evidence supporting a role for *CtARF4* in safflower floral development. Perturbation of *CtARF4* expression, through either overexpression or gene silencing, led to consistent alterations in inflorescence-related traits. *CtARF4* was localized to the nucleus, enhanced *CtMADS24* promoter activity in reporter assays, and physically interacted with *CtIAA9*, supporting its function as a transcriptional regulator.

Importantly, empty vector controls for both gene silencing (pTRV1-pTRV2) and overexpression (pGreenII-SK-62-Flag) were included, and the corresponding data are provided in Supplementary Figures S1 and S4. The absence of significant differences between these controls and WT plants supports the specificity of *CtARF4*-mediated effects. The observed inverse relationship between *CtARF4* expression and endogenous IAA levels may reflect feedback regulation within the auxin signaling pathway, although the underlying mechanism requires further investigation.

Despite these findings, the precise role of *CtIAA9* in safflower floral development remains unclear. Therefore, we propose a working model in which *CtARF4* may regulate inflorescence development through auxin-related signaling, potentially involving both *CtMADS24* and *CtIAA9*. Further studies will be required to elucidate how auxin dynamics and *CtIAA9*-mediated regulation influence *CtARF4* activity at the *CtMADS24* promoter.

## 4. Materials and Methods

### 4.1. Identification, Phylogenetic Analysis, and Target Prediction of CtARF Transcription Factors in Safflower

The safflower genome sequence was used to identify ARF family members. ARF protein sequences of *Arabidopsisthaliana* were retrieved from The *Arabidopsis* Information Resource (TAIR, https://www.arabidopsis.org accessed on 5 June 2023), and sunflower (*Helianthus annuus*) ARF sequences were obtained from the NCBI database (https://www.ncbi.nlm.nih.gov accessed on 6 June 2023). These sequences were using the NCBI BLAST web server (https://blast.ncbi.nlm.nih.gov/Blast.cgi, accessed on 6 June 2023) against the safflower genome database.

A phylogenetic tree was constructed using MEGAX (v10.2.6) with the neighbor-joining (NJ) method and 1000 bootstrap replicates, based on protein sequences of *CtARFs* from safflower transcriptome data together with ARF homologs from *Arabidopsis thaliana* and *Helianthus annuus*. The *CtARF* genes were classified according to the established grouping patterns of the *Arabidopsis* and *Helianthus ARF* gene families. The resulting tree was visualized and annotated using the online tool Evolview (https://www.evolgenius.info/evolview/#/, accessed on 6 June 2023) [36].

Conserved protein motifs were identified from the full-length protein sequences of *CtARFs* using the MEME suite (http://meme-suite.org/tools/meme accessed on 7 June 2023) with the following parameters: maximum number of motifs = 10, minimum width = 6, and maximum width = 200. Gene structure features, including the distribution of introns and exons, were analyzed using the GSDS web server (http://gsds.cbi.pku.edu.cn/ accessed on 9 June 2023) by comparing the genomic and coding DNA sequences of each candidate *CtARF* gene.

To predict homologous TF–promoter regulatory relationships in safflower (*Carthamus tinctorius*), we integrated homology-based mapping with motif scanning. TF annotations and non-redundant position weight matrices (PWMs) for *Arabidopsis thaliana* were retrieved from PlantTFDB (v5.0) [24] and JASPAR (2026) [25]. Promoter regions, defined as the 2000 bp sequences upstream of the start codon, were extracted from the safflower genome. Orthologous TF pairs between *A. thaliana* and *C. tinctorius* were identified using blastp based on the bidirectional best hit (BBH) strategy with an E-value threshold of 1 × 10^−5^. Subsequently, motif scanning was performed using FIMO [37] from the MEME [38] Suite (v5.5.0) to map *Arabidopsis* PWMs onto safflower orthologous promoters, applying a significance threshold of *p* < 1 × 10^−4^. The final regulatory network was constructed using Python 3.9 by intersecting the orthology data with significant motif-binding sites, followed by filtering to retain only annotated TFs in the safflower genome.

### 4.2. Transcriptome Data Processing and Expression Analysis of CtARF Genes

Transcriptome datasets used in this study were obtained from previously published RNA-seq data of safflower (BioProject accession: PRJNA909037). The dataset includes transcriptomic profiles from four flower developmental stages (bud stage, initial flowering stage, full bloom stage, and fading stage) as well as five tissues (root, stem, leaf, flower, and seed). All plant materials were derived from the safflower cultivar Jihong No.1.

Raw sequencing reads were subjected to quality control using FastQC software(v0.11.9). Low-quality reads and adapter sequences were removed using Trimmomatic to obtain high-quality clean reads. The filtered reads were then aligned to the safflower reference genome using HISAT2(v 2.2.1).

Gene expression levels were quantified using StringTie (v2.2.1)and normalized as fragments per kilobase of transcript per million mapped reads (FPKM). Expression patterns of *CtARF* genes across different tissues and developmental stages were visualized using heatmaps generated with the pheatmap package in R(v 4.2.0) [39].

### 4.3. RNA Extraction and Quantitative Real-Time PCR (qRT-PCR) Analysis

Total RNA was extracted from safflower tissues using the FastPure Plant Total RNA Isolation Kit (Vazyme, Nanjing, China) following the manufacturer’s instructions. RNA purity and concentration were determined using a NanoDrop 2000 spectrophotometer (Thermo Fisher Scientific, Wilmington, DE, USA).

First-strand cDNA was synthesized from 1 μg of total RNA using the MonScript™ RTIII All-in-One Mix with dsDNase (Monad, Suzhou, China; Cat. No. MR05201). The synthesized cDNA was stored at −20 °C until use.

Quantitative real-time PCR (qRT-PCR) was performed using a Stratagene MX3000P Real-Time PCR System with reagents from Monad Biotechnology (Suzhou, China). Each 20 μL reaction mixture contained 2.0 μL of diluted cDNA template, 10 μL of 2× qPCR master mix, 0.2 μM of each forward and reverse primer, 0.2 μL ROX reference dye (100×), and RNase-free water to volume.

The safflower gene CtEF1α was used as the internal reference gene (Forward: TCAGCATTGTCGTCATCGGA; Reverse: ACGTTCGATCACACGCTTGTC). The PCR cycling conditions were as follows: 95 °C for 30 s, followed by 40 cycles of 95 °C for 5 s and 60 °C for 30 s.

Relative gene expression levels were calculated using the 2^−ΔΔCt^ method. Three independent biological replicates were analyzed, and each biological replicate included three technical replicates.

### 4.4. Statistical Analysis

All experiments were performed with at least three independent biological replicates unless otherwise specified. Data are presented as mean ± standard deviation (SD). Statistical analyses were conducted using SPSS 26.0 (IBM, New York, NY, USA). Differences between two groups were evaluated using Student’s *t*-test, whereas comparisons among multiple groups were performed using one-way analysis of variance (ANOVA) followed by Tukey’s multiple comparison test. Differences were considered statistically significant at *p* < 0.05.

### 4.5. Subcellular Localization

Subcellular localization of *CtARF4* was analyzed by transiently expressing a *CtARF4*-DsRed fusion protein in tobacco leaves. The recombinant plasmid pGDR-*CtARF4* and the empty vector pGDR, used as a negative control, were introduced into *Agrobacterium tumefaciens* strain GV3101 (pSoup-p19). *Agrobacterium* cultures were adjusted to an OD600 of 0.8 and infiltrated into the abaxial side of *Nicotiana benthamiana* leaves with a needleless syringe [40]. After incubation for 48 h under dark conditions, the fluorescence signal was observed and captured using a laser scanning confocal microscope (Leica Microsystems, Wetzlar, Germany).

### 4.6. Transient Over-Expression and Virus-Induced Gene Silencing (VIGS) of CtARF4 in Safflower

To investigate the function of *CtARF4* in safflower floral development, both virus-induced gene silencing (VIGS) and transient overexpression approaches were employed.

For transient overexpression, the full-length coding sequence of *CtARF4* was cloned into the pGreenII 62-SK-Flag vector under the control of the CaMV 35S promoter to generate the construct pGreenII 62-SK-Flag-*CtARF4*, while the empty pGreenII 62-SK-Flag vector was used as a negative control. For gene silencing, a gene-specific fragment of *CtARF4* was inserted into the pTRV2 vector to generate pTRV2-*CtARF4*, and the empty pTRV2 vector served as the control. All constructs were introduced into Agrobacterium tumefaciens strain GV3101 (pSoup-p19).

Agrobacterium cultures were grown to an OD600 = 0.8, collected by centrifugation, and resuspended in infiltration buffer (10 mM MgCl_2_, 10 mM MES, and 200 μM acetosyringone). After incubation in the dark for 2 h at room temperature, bacterial suspensions were used for infiltration. For VIGS, cultures carrying pTRV1 and either pTRV2 or pTRV2-*CtARF4* were mixed at a 1:1 ratio prior to infiltration.

Bacterial suspensions were infiltrated into safflower flower buds at the early developmental stage using a needleless syringe. Plants were subsequently grown under normal conditions until flowering. Phenotypic traits, including flowering time, floret number, and floret length, were recorded.

A total of 15 independent overexpression lines and 15 independent silencing lines were analyzed, and three biological replicates were used for quantitative measurements. The efficiency of *CtARF4* overexpression and silencing, as well as the expression of *CtMADS24*, were verified by qRT-PCR as described in Section 4.3.

### 4.7. Yeast One-Hybrid Assay

The coding sequence of *CtARF4* and the promoter fragment of *CtMADS24* (proCtMADS24) were cloned into the pGADT7 (prey) and pHIS2 (bait) vectors, respectively. The resulting constructs were co-transformed into the yeast strain Y187 according to the manufacturer’s instructions.

To assess the interaction between *CtARF4* and the *CtMADS24* promoter, transformed yeast cells were serially diluted (10^−1^–10^−6^) and spotted onto SD/-Leu/-Trp/-His (SD/-LTH) selective medium. Plates were incubated at 30 °C for 3–4 days, and yeast growth on selective medium was used as an indicator of protein–DNA interaction.

### 4.8. Dual-Luciferase Reporter Assay

The recombinant vectors pGreenII 0800-Luc-proCtMADS24 (reporter) and pGreenII 62-SK-*CtARF4* (effector) were constructed and introduced into *Agrobacterium tumefaciens* strain GV3101 (pSoup-p19). *Agrobacterium cultures* were adjusted to an OD600 of 0.8, mixed in a 1:1 ratio, kept in the dark at room temperature for 2 h, and then infiltrated into the abaxial side of three-week-old *Nicotiana benthamiana* leaves using a needleless syringe. After incubation in darkness for 24 h, the plants were transferred to a growth chamber under normal light and temperature conditions for an additional 24 h. Luminescence signals were captured and analyzed using a plant live imaging system at 48 h post-infiltration.

### 4.9. Yeast Two-Hybrid Assay

The coding sequences of *CtARF4* and *CtIAA9* were cloned into the pGBKT7 (DNA-binding domain vector) and pGADT7 (activation domain vector), respectively, to generate the bait and prey constructs. The resulting plasmid pairs were co-transformed into the yeast strain Y2HGold. Transformed yeast cells were cultured in SD/–Leu/–Trp (DDO) medium and then subjected to serial dilutions (10^−1^ to 10^−4)^. Aliquots of each dilution were spotted onto SD/–Leu/–Trp (DDO) control medium and SD/–Leu/–Trp/–His/–Ade (QDO) selective medium supplemented with X-α-gal. The plates were incubated at 30 °C for 3–4 days, judging the transcriptional activation activity of *CtARF4* by the presence of blue colonies [41].

### 4.10. Bimolecular Fluorescence Complementation (BiFC) Assay

The recombinant vectors pxy104-*CtARF4* and pxy106-*CtIAA9* were introduced into *Agrobacterium tumefaciens* strain GV3101 (pSoup-p19). *Agrobacterium* cultures were adjusted to an OD600 = 0.8, and the two bacterial suspensions were mixed in a 1:1 ratio. The empty vectors pxy104 and pxy106 were used as negative controls and were introduced into *Agrobacterium* in the same manner. The bacterial suspensions were infiltrated into the abaxial side of three-week-old *Nicotiana benthamiana* leaves with a needleless syringe. After 24 h of dark incubation, plants were transferred to a growth chamber under normal conditions. YFP fluorescence signals were detected using a confocal microscope (Leica TCS SP8) at 48 h post-infiltration.

### 4.11. Plant Treatment for Validating the Effect of Auxin on Gene Expression

Safflower buds at the same developmental stage were divided into three groups: the first group was sprayed with water (serving as the control); the second group was treated with 50 μM NAA; and the third group was treated with 50 μM NPA [15]. The spraying was repeated every three days. After three treatments, plant materials were harvested, immediately frozen in liquid nitrogen, and ground into a fine powder. Total RNA was extracted using the FastPure Plant Total RNA Isolation Kit. First-strand cDNA was synthesized from the extracted RNA using the MonScript™ RTIII All-in-One Mix with dsDNase. Finally, the expressions of three genes, *CtARF4*, *CtMADS24*, and *CtIAA9*, were analyzed by qRT-PCR.

### 4.12. Endogenous IAA Quantification

Endogenous indole-3-acetic acid (IAA) levels were quantified using a commercial Plant Auxin (IAA) Detection Kit (KIRbio, Shanghai, China; Cat. No. K8-190543) following the manufacturer’s protocol. Approximately 150 mg of fresh flower tissue was collected from plants at the same developmental stage, immediately frozen in liquid nitrogen, and ground to a fine powder. The tissue powder was homogenized in 1 mL of the extraction buffer provided in the kit and incubated on ice for 30 min. After centrifugation at 12,000× *g* for 10 min at 4 °C, the supernatant was collected for analysis. When necessary, samples were appropriately diluted with extraction buffer to ensure that absorbance values fell within the linear range of the standard curve.

Absorbance was measured at 450 nm using a microplate reader. A standard curve was generated in each assay using the kit-provided standards, and IAA concentrations were calculated by linear regression (R^2^ ≥ 0.99). IAA content was expressed as ng per g fresh weight.

Three independent biological replicates were analyzed, each consisting of pooled flower tissue from at least three individual plants. For each biological replicate, measurements were performed in triplicate. Statistical analysis was conducted using Student’s *t*-test, and differences were considered significant at *p* < 0.05.

## 5. Conclusions

This study elucidated the role of the *CtARF4* transcription factor in safflower flower development. A total of 25 *CtARF* members were identified across the safflower genome. Phylogenetic analysis was conducted to classify these members, and the prediction of transcription factor binding sites within their promoter regions was performed. Expression profiling of *CtARF* genes at different developmental stages and floral tissues suggested their potential involvement in safflower flower development.

Subcellular localization confirmed that *CtARF4* is expressed in the plant nucleus. Yeast one-hybrid and luciferase complementation assays demonstrated that *CtARF4* binds to the *CtMADS24* promoter. Moreover, yeast two-hybrid and bimolecular fluorescence complementation (BiFC) experiments revealed a protein–protein interaction between *CtARF4* and *CtIAA9*.

Functional characterization through virus-induced gene silencing and transient overexpression of *CtARF4* further verified its role in regulating flowering time and floret development in safflower. These findings provide valuable resources for future molecular breeding efforts aimed at developing safflower varieties with early flowering and improved petal yield.

## Data Availability

No new data were created or analyzed in this study.

## Supplementary Materials

The following supporting information can be downloaded at https://www.mdpi.com/article/10.3390/plants15071110/s1, Figure S1: Identification of *CtARF4*-silenced and overexpression lines based on relative expression analysis. Figure S2: Phenotypic analysis of floral morphology in *CtARF4* transgenic plants, including capitulum, individual florets, and dissected florets. Figure S3: Expression profiles of *CtMADS24* and *CtIAA9* during different stages of safflower flower development. Figure S4: Quantitative analysis of floral traits in *CtARF4* transgenic plants, including floret length, days to anthesis, number of florets per capitulum, and capitulum diameter.

## Abbreviations

The following abbreviations are used in this manuscript:

ARF	Auxin response factors
AP	APETALA
IAA	indole-3-acetic acid
DBD	DNA-binding domain
MR	middle region
AD	activation domain
RD	repression domain
CTD	C-terminal dimerization domain
MAD	MADS-box transcription factor family
