# Supplementary material for: CtARF4 Regulates Inflorescence Development Through Transcriptional Regulation of CtMADS24 in Safflower
Source: Plants (Basel). 2026 Apr 3;15(7):1110. doi: 10.3390/plants15071110 (PMC13074928; doi:10.3390/plants15071110)
Supplement: Supplementary file 1 [file plants-15-01110-s001.zip › plants-4173892-supplementary.pdf]

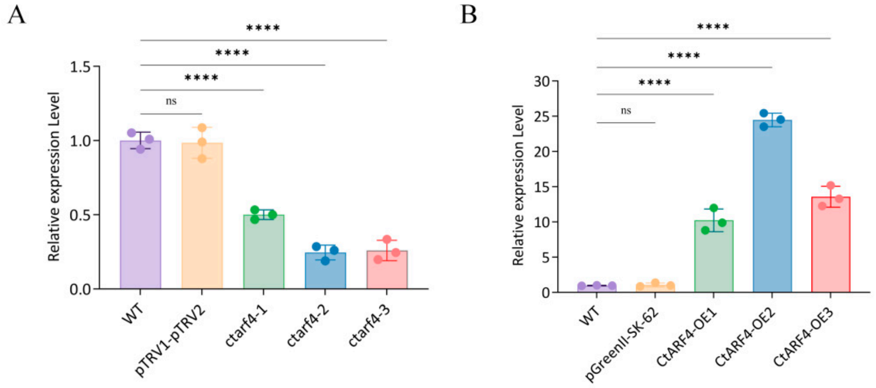

Supplementary Figure S1. Identification of CtARF4-silenced and overexpression lines by relative expression analysis.

(A) Relative expression levels of CtARF4 in wild-type (WT), empty vector control for gene silencing (pTRV1-pTRV2), and CtARF4-silenced lines (ctarf4-1, ctarf4-2, and ctarf4-3).

(B) Relative expression levels of CtARF4 in wild-type (WT), empty vector control for overexpression (pGreenII-SK-62-Flag), and CtARF4-overexpression lines (CtARF4-OE1, CtARF4-OE2, and CtARF4-OE3).Data are presented as mean ± SD from three biological replicates (n = 3). Statistical significance was determined by one-way ANOVA followed by multiple comparisons. Differences are indicated as follows: ** $p < 0.01$, **** $p < 0.0001$; ns, not significant.

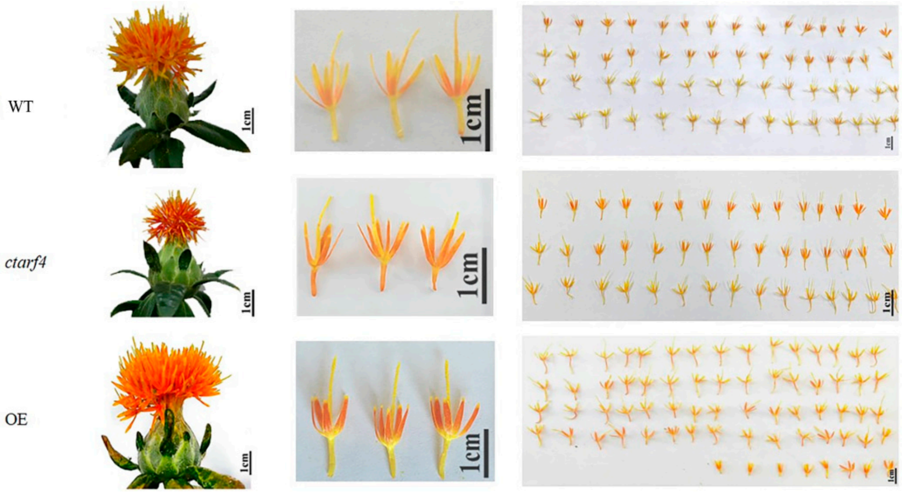

Supplementary Figure S2. Phenotypic analysis of floral morphology in CtARF4 transgenic plants.

Representative images of capitulum (left), individual florets (middle), and dissected florets (right) from control plants (control), CtARF4-silenced plants (ctarf4), and CtARF4-overexpression plants (OE). Scale bars = 1 cm.

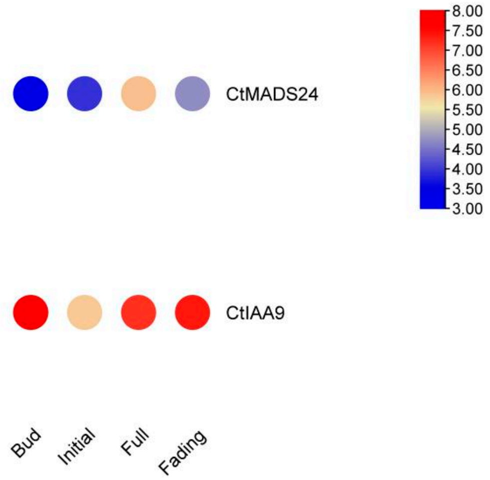

Supplementary Figure S3. Expression profiles of CtMADS24 and CtIAA9 during different stages of safflower flower development.

Relative expression levels of CtMADS24 and CtIAA9 were analyzed at four developmental stages, including bud, initial flowering, full flowering, and fading stages. The color scale represents expression levels, with blue indicating low expression and red indicating high expression.

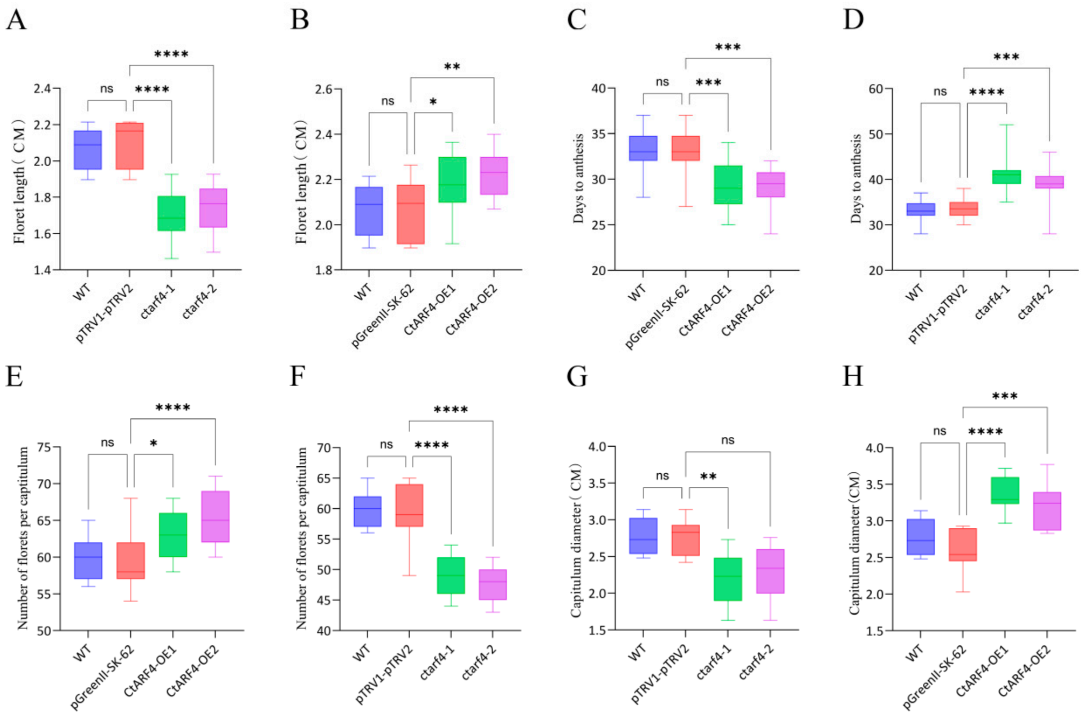

Supplementary Figure S4. Analysis of floral traits in CtARF4 transgenic plants.

(A,B) Floret length; (C,D) days to anthesis; (E,F) number of florets per capitulum; and (G,H) capitulum diameter in wild-type (WT), empty vector control for gene silencing (pTRV1-pTRV2), CtARF4-silenced lines (ctarf4-1 and ctarf4-2), empty vector control for overexpression (pGreenII-SK-62), and CtARF4-overexpression lines (CtARF4-OE1 and CtARF4-OE2).

Data are presented as box plots showing the median, interquartile range, and full data distribution *n*=15. Statistical

significance was determined by one-way ANOVA test. Comparisons were performed between WT and the corresponding empty vector controls, and between empty vector controls and the corresponding CtARF4-silenced or overexpression lines. Differences are indicated by asterisks as follows: * $p < 0.05$, ** $p < 0.01$, *** $p < 0.001$, **** $p < 0.0001$; ns, not significant.
